# Cost-effective detection of genome-wide signatures for 2,4-D herbicide resistance adaptation in red clover

**DOI:** 10.1038/s41598-019-55676-9

**Published:** 2019-12-27

**Authors:** Juliana Benevenuto, Mehul Bhakta, Daniel A. Lohr, Luís Felipe V. Ferrão, Marcio F. R. Resende, Matias Kirst, Kenneth Quesenberry, Patricio Munoz

**Affiliations:** 1 Blueberry Breeding and Genomics Laboratory, Department of Horticultural Sciences, IFAS, University of Florida, Gainesville, FL USA; 2 Forage Breeding and Genetics Laboratory, Department of Agronomy, IFAS, University of Florida, Gainesville, FL USA; 3 Sweet Corn Genomics and Breeding, Department of Horticultural Sciences, IFAS, University of Florida, Gainesville, FL USA; 4 School of Forest Resources and Conservation, IFAS, University of Florida, Gainesville, FL USA

**Keywords:** Genome-wide association studies, Population genetics, Natural variation in plants, Plant breeding, Plant evolution, Next-generation sequencing, Agricultural genetics

## Abstract

Herbicide resistance is a recurrent evolutionary event that has been reported across many species and for all major herbicide modes of action. The synthetic auxinic herbicide 2,4-dichlorophenoxyacetic acid (2,4-D) has been widely used since the 1940s, however the genetic variation underlying naturally evolving resistance remains largely unknown. In this study, we used populations of the forage legume crop red clover (*Trifolium pratense* L.) that were recurrently selected for 2,4-D resistance to detect genome-wide signatures of adaptation. Four susceptible and six derived resistant populations were sequenced using a less costly approach by combining targeted sequencing (Capture-Seq) with pooled individuals (Pool-Seq). Genomic signatures of selection were identified using: (i) pairwise allele frequency differences; (ii) genome scan for overly differentiated loci; and (iii) genome‐wide association. Fifty significant SNPs were consistently detected, most located in a single chromosome, which can be useful for marker assisted selection. Additionally, we searched for candidate genes at these genomic regions to gain insights into potential molecular mechanisms underlying 2,4-D resistance. Among the predicted functions of candidate genes, we found some related to the auxin metabolism, response to oxidative stress, and detoxification, which are also promising for further functional validation studies.

## Introduction

Overreliance on herbicides to minimize weed competition and maximize crop yield imposes strong selective pressure toward herbicide resistance^1,2^. The emergence of resistant phenotypes in weeds is widespread across taxonomic groups (254 species), geographic regions (70 countries), and for all major chemical classes of herbicides (23 out of 26 known modes of action)^3^. While herbicide resistance in weeds is a threat to agriculture^4^, it is a desirable trait in crop plants^5,6^. Herbicide resistant crop varieties have been obtained by transgenic and traditional breeding methods aiming to improve herbicide selectivity, expand weed control spectrum, and minimize crop injury^7,8^.

The chemical 2,4-dichlorophenoxyacetic acid (2,4-D) was the first synthetic herbicide developed for dicot weed control in cereal fields, and has remained one of the most commonly used since the 1940s. Despite being widely used for more than 70 years, few species have naturally evolved resistance to 2,4-D^6,9^. To our knowledge, there are 16 cases of 2,4-D resistance in dicot weeds and two resistant crops obtained by traditional breeding reported to date^10–12^. The absence of widespread resistance adaptation to 2,4-D is likely due to its complex mode of action, with many avenues of functional redundancy and fitness penalties of mutations in its pathways^13^. 2,4-D is a synthetic small molecule structurally and functionally analogous to the natural auxin indole-3-acetic acid (IAA) and induces the same type of responses as this phytohormone. Both IAA and 2,4-D are actively transported into plant cells via common influx (AUX1/LAX family) and efflux (PIN and ABCB families) carrier proteins, and both can bind to auxin receptor protein TIR1 or its homologs AFBs, leading to auxin-responsive gene expression^14,15^. However, 2,4-D has a long-lasting effect, since it is less prone to degradation and inactivation than IAA^16,17^. At herbicidal concentrations, 2,4-D promotes an imbalance in auxin homeostasis, leading to a continued expression of auxin-responsive genes; increased synthesis of ethylene, abscisic acid (ABA), and reactive oxygen species (ROS); abnormal growth; tissue desiccation and decay; necrosis; and finally, plant death^14,15,18–20^.

Given the multiple and essential roles of auxin in plant growth and development, mutants resistant to 2,4-D are expected to also exhibit altered response to the natural plant hormone and, consequently, suffer fitness tradeoffs and abnormalities^21,22^. Most of the adaptive mechanisms to auxinic herbicides are generally reported as non-target-site resistance (NTSR)^15,16,23^. Some evidence of NTSR mechanisms for 2,4-D included: reduced herbicide uptake/absorption^24^, reduced translocation^16,25,26^, and detoxification by enhanced metabolism^27,28^. However, the most comprehensive study of a naturally-evolved auxinic herbicide resistance mechanism was conducted very recently for the dicamba-resistant weed kochia (*Kochia scoparia* L.), where a target site resistance mechanism was detected. Two nucleotides mutation at the auxin co-receptor IAA16 conferred resistance to dicamba, and it was also speculated to endow cross-resistance to 2,4-D^22^. A recent transcriptomics study also provided insights on the molecular pathways potentially leading to 2,4-D resistant in wild radish weed (*Raphanus raphanistrum* L.)^29^. However, distinct genetic and molecular basis are likely involved in each resistant species or population, and genomic adaptation studies for 2,4-D resistance have largely lagged behind^23^.

Red clover (*Trifolium pratense* L.) is a forage legume crop that has been bred for 2,4-D tolerance because this herbicide is one of the most commonly used for weed control in grass pastures^12^. The tolerant cultivar ‘FL24D’ was developed at the University of Florida after six cycles of recurrent mass selection for tolerance to 2,4-D^12^. When susceptible and tolerant plants are sprayed with 2,4-D, the former ones die, while the tolerant plants show strong damage but regrow from the crown meristematic tissue. Asymptomatic new growth after herbicide application is also reported in 2,4-D resistant weeds^16^. Under unsprayed conditions, ‘FL24D’ does not exhibit impaired traits compared to susceptible cultivars, but it differs in precocity, having earlier growth in spring than other cultivars^30^.

In this study, we aimed to identify the genomic regions underlying the evolution of 2,4-D resistance in recurrently selected populations of red clover. These results are useful for further molecular breeding of red clover and to narrow down candidate genes potentially involved in herbicide resistance. We hypothesized that the selective pressure imposed by 2,4-D treatment over the breeding cycles has led to an increase in the frequency of alleles contributing toward herbicide resistance. To estimate population allele frequency and detect genomic signatures of adaptive divergence and phenotypic variation at a lower cost, the pooled sequencing (Pool-Seq) of individuals strategy has been extensively employed^31–35^. In this study, we used a combination of Pool-Seq and targeted sequencing approach (Capture-Seq) from six resistant and four susceptible red clover populations. In order to identify significant genomic signatures of adaptive differentiation between resistant and susceptible populations, we used three different approaches: (i) comparisons of pairwise allele frequency differences; (ii) genome scan for overly differentiated loci; and (iii) genome‐wide association using resistance and susceptible phenotypes as a population‐specific covariable. Variants showing concordant results across methods were further investigated for their putative functional significance.

## Results

### SNP calling and genetic relationship among samples

**Figure 1:**
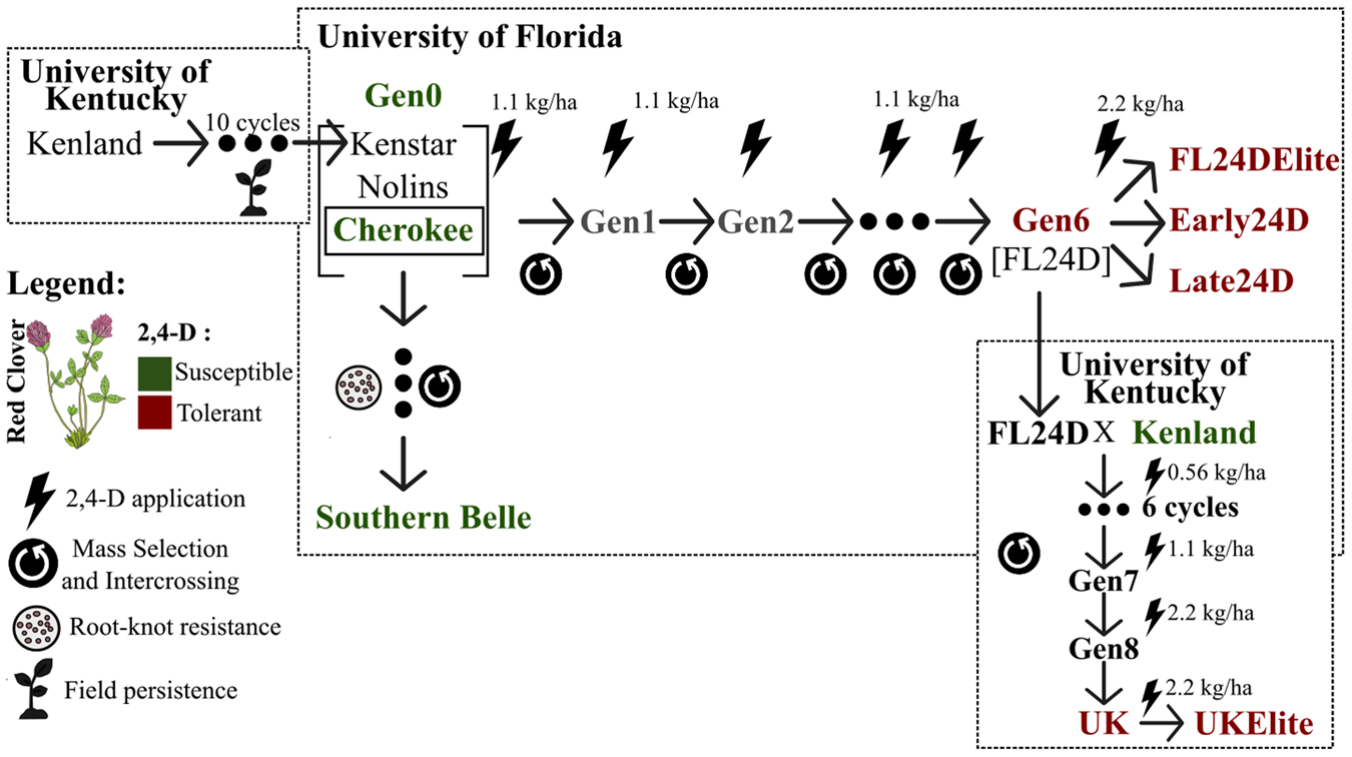
Breeding scheme of the plant material used in this study. The six resistant and four susceptible synthetic cultivars are shown in red and green, respectively. The recommended rate of 2,4-D application is 1.1 kg ha^−1^ of active ingredient for reference.

In this study, six resistant and four susceptible pools of individuals from synthetic red clover populations were sequenced using a capture-seq approach (Fig. 1 and Table 1). A total of 6,407,589 SNPs were detected at first. After applying stringent filtering criteria, we selected 11,768 SNPs that were biallelic, present across all ten pools (no missing data), uniquely mapped (read mapping quality greater than 20), with minimum and maximum depth of coverage of 40 and 400, respectively. SNPs were also well distributed throughout the seven red clover chromosomes.

**Table 1:** Number of pooled individuals, number of sequenced reads, and sequencing platform used for each sample.

Pooled Samples	Resistant (R) or Susceptible (S)	Number of Individuals	Raw Read Count	Illumina Platform	SRA Accession Number
Gen0	S	20	128,762,194	NextSeq 500	SRR8157540
Cherokee	S	20	117,929,104	NextSeq 500	SRR8157541
Southern Belle	S	26	144,553,898	NextSeq 500	SRR8157538
Gen6	R	48	100,041,080	NextSeq 500	SRR8157539
Kenland	S	27	34,717,334	HiSeq3000	SRR8157543
FL24DElite	R	24	39,236,182	HiSeq3000	SRR8157536
Early24D	R	28	37,229,808	HiSeq3000	SRR8157537
Late24D	R	27	41,948,810	HiSeq3000	SRR8157534
UK	R	30	35,126,246	HiSeq3000	SRR8157535
UKElite	R	26	38,124,186	HiSeq3000	SRR8157542

**Figure 2:**
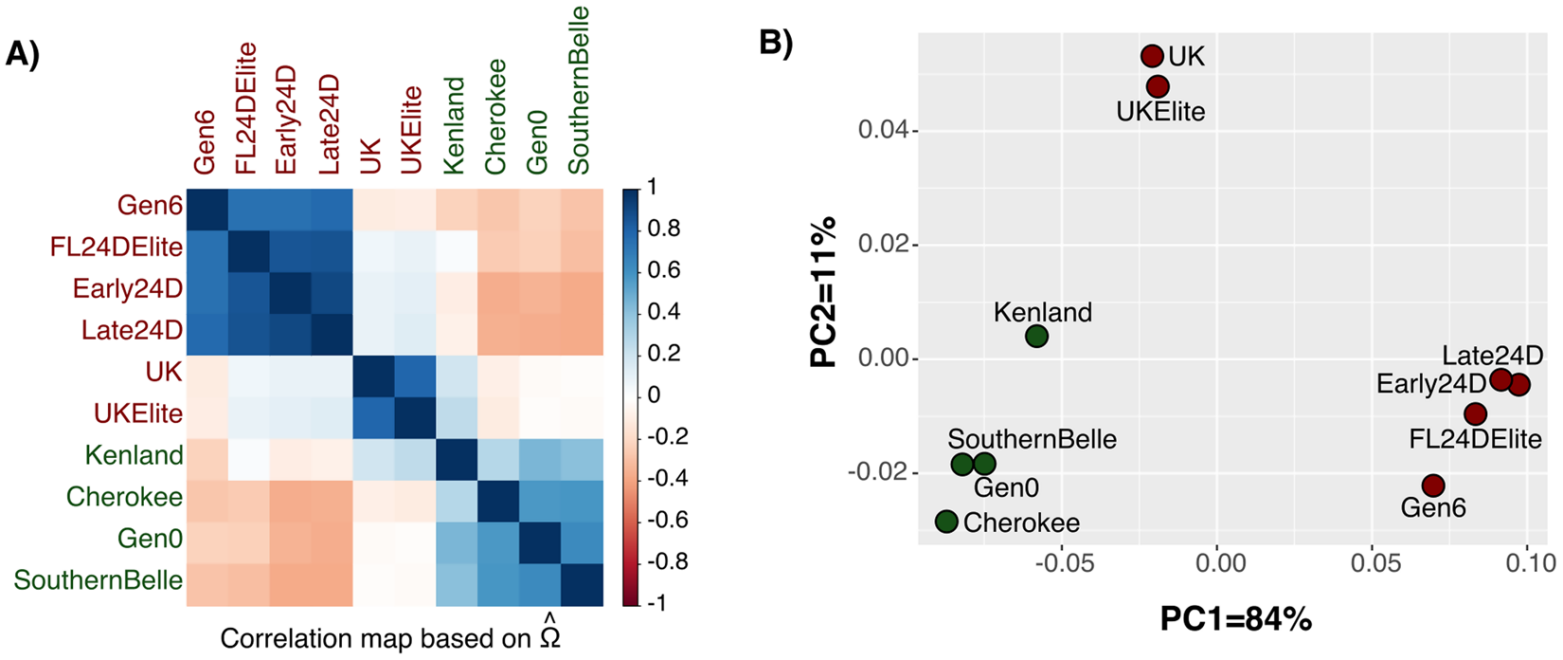
Genetic relationship among the ten synthetic red clover cultivars (pooled individuals). (**A**) Correlation plot and (**B**) Principal Component Analysis (PCA) based on the covariance matrix Ω estimated under the Baypass core model. Resistant and susceptible synthetic cultivars are shown in red and green, respectively.

The estimated covariance matrix of allele frequencies (Ω) based on read counts of 11,768 polymorphic sites was used to quantify the genetic relationship among pools (Fig. 2). The correlation plot (Fig. 2A) and PCA (Fig. 2B) clusters reflected the expected relationship between synthetic cultivars from the recorded pedigree information (Fig. 1).

### Genome-wide signatures of selection

**Figure 3:**
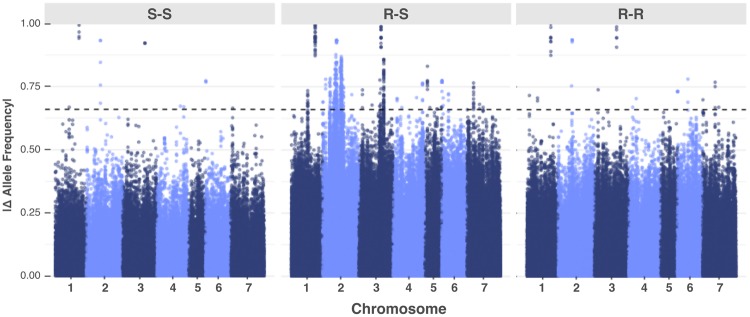
Overlap of pairwise allele frequency differences among Susceptible-Susceptible (S-S), Resistant-Susceptible (R-S), and Resistant-Resistant (R-R) comparisons. Absolute values of allele frequency from 11,768 SNPs were plotted. The threshold was determined as the 99.9^th^ percentile of the allele frequency difference distribution across all values.

We performed a preliminary screen for differences in raw allele frequency among pools. The pairwise comparisons showed consistent outliers when resistant and susceptible (R-S) pools were compared, which were not detected in resistant-resistant (R-R) or susceptible-susceptible (S-S) contrasts (Figs. 3 and S1). Most SNPs showing high allele frequency differences between R-S were detected at chromosome 2.

**Figure 4:**
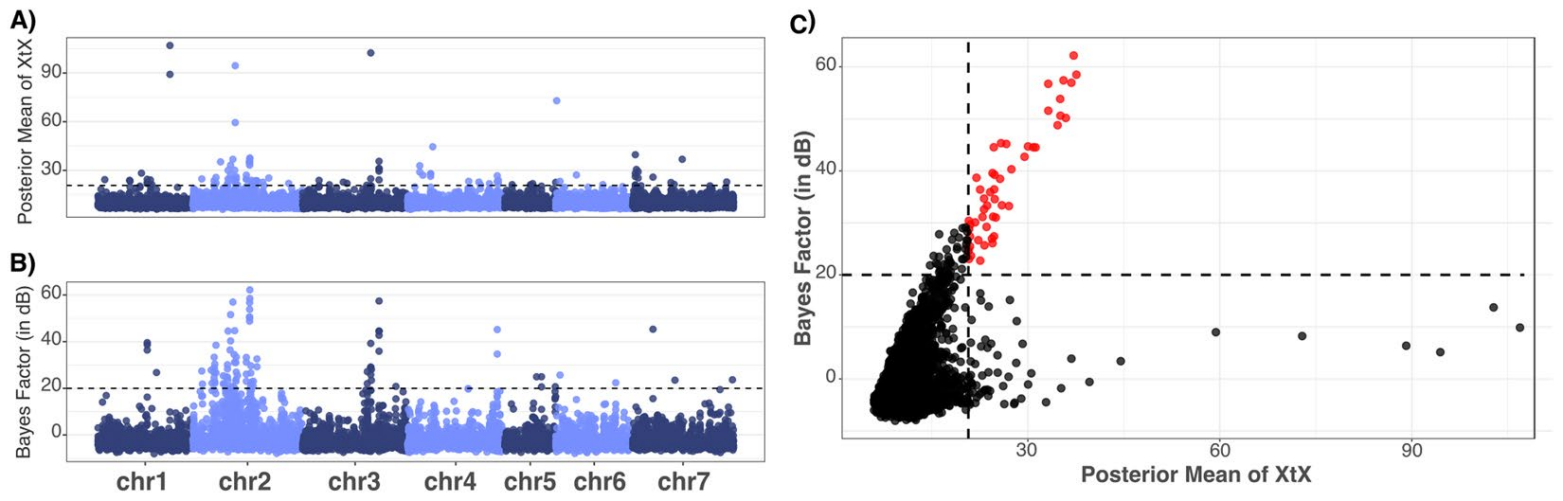
Genomic signatures of 2,4-D resistance adaptation. (**A**) Genomic scan for overly differentiated loci based on XtX statistics estimated under the Baypass core model. The dashed line represents the 0.1% POD significance threshold (XtX = 20.70). (**B**) Genome-wide association with the herbicide resistance/susceptibility covariable under the Baypass AUX model. The y-axis indicates the Bayes Factor expressed in deciban units (dB). The dashed line represents the 20-dB significance threshold. In both plots, the x-axes indicate the position of the 11,768 SNPs along the seven red clover chromosomes (“chr”). (**C**) The XtX genetic differentiation value as a function of the Bayes Factor (BF) in deciban (dB) of the association with the herbicide resistance/susceptibility covariable. The vertical and horizontal dashed lines represent the respective thresholds. Red dots represent the 50 outliers considering both XtX and BF values.

To formally detect significant signatures of selection based on the allele frequency differences among the ten pools, we used two distinct and robust Bayesian frameworks, correcting for the relationship among pools and sampling noise. First, a genomic scan for overly differentiated SNPs was performed based on the XtX measure, which is analogous to F_*ST*_, but explicitly accounts for the relationship among populations and sampling noise in pooled samples. A pseudo‐observed data set (POD) was simulated to estimate the posterior predictive distribution of the XtX statistics under neutrality, providing the threshold for detecting overly differentiated SNPs among populations. The estimate of Ω on the POD neutral simulation was close to the matrix estimated on the original data set (FMD = 0.39), indicating that the POD can be used to define the significance threshold on XtX analysis. In total, 107 SNPs were found as outliers at the 0.1% POD significance threshold. Most of the significant outliers (47) were found at chromosome 2 (Fig. 4A). Second, analyses of association were conducted using the resistance/susceptible phenotype as a categorical pool-specific covariable. In total, 88 SNPs were significant at 20-dB threshold, with 59 identified at chromosome 2 (Fig. 4B). Interestingly, some overly differentiated SNPs (high XtX value) were not associated with the herbicide resistant/susceptible phenotype (small Bayes Factor), indicating the presence of other selective pressures (Fig. 4C). Considering common outliers from both approaches, we detected 50 significant SNPs (Fig. 4C), providing consistent evidence for selection at these genomic regions. Moreover, we selected the most significant variant in each chromosome for individual Sanger sequencing and SNP validation. To this end, we used an independent set of resistant and susceptible individuals from ‘FL24D’ and ‘Southern Belle’ respectively. SNPs at chromosome 1, 2, and 3 were also significant at Fisher’s exact test, providing further empirical support for their presence and association (Supplementary Table S2 and Fig. S2).

### Candidate genes underlying significant SNPs

**Figure 5:**
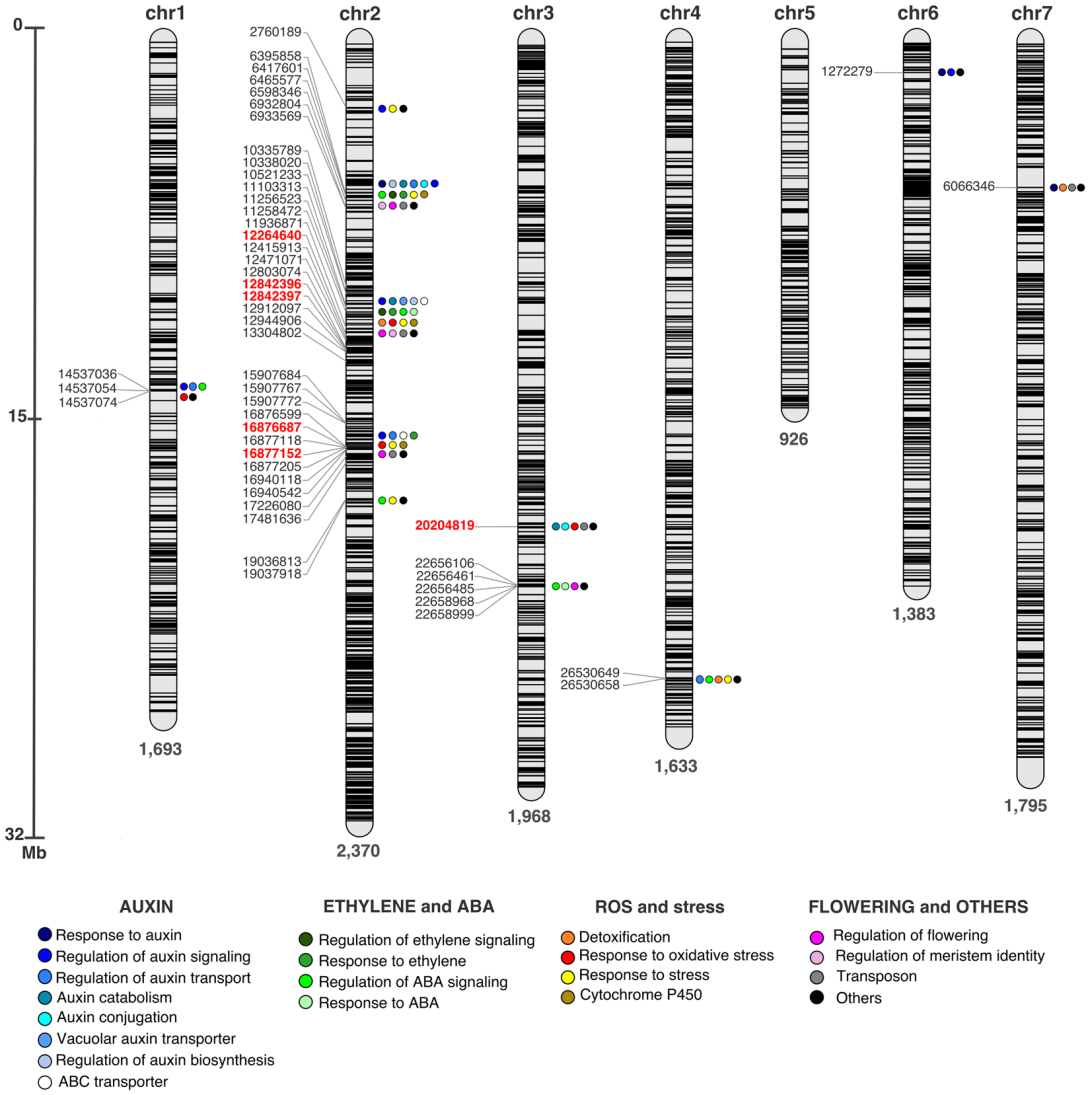
Distribution of SNPs and annotation of genes flanking significant SNPs. Distribution of 11,768 filtered SNPs across the seven chromosomes (“chr”) of red clover ‘Milvus B’ genome, localization of the 50 significant SNPs for herbicide resistance adaptation, and relevant functional classification of candidate genes within a ±100 kb window flanking significant SNPs. Numbers below each chromosome indicate the total number of SNPs. SNPs highlighted in red caused missense mutations.

To gain insights into the potential functional significance of the outlier loci detected by two distinct approaches, we retrieved the annotation of the protein-coding genes flanking the 50 SNPs in the red clover genome. Most of the significant SNPs (37) were located at chromosome 2, followed by six SNPs at chromosome 3, three at chromosome 1, two at chromosome 4, and one each at chromosome 6 and 7 (Fig. 5 and Table 2). Twenty SNPs were located in protein coding sequences, with six of them causing missense mutations. Among the remaining SNPs, 20 were located at introns, seven at untranslated regions (UTR), and three at intergenic regions (Supplementary Table S1). Based on sequence homology of candidate genes surrounding significant SNPs, we detected several candidate genes with putative orthologs known to be directly involved in auxin homeostasis, such as regulators of auxin response (cullin-associated NEDD8-dissociated protein 1; NEDD8-conjugating enzyme Ubc12; BTB/POZ domain-containing protein NPY4; protein SHI RELATED SEQUENCE 1; transcription factor MYB44 and MYB61; receptor-like kinase TMK4, protein PIN-LIKES 7; auxin-responsive protein IAA20; auxin-responsive protein SAUR32), transport (serine/threonine-protein kinase D6PKL1; VAN3-binding protein; protein WALLS ARE THIN 1; protein SHOOT GRAVITROPISM 5), conjugation (indole-3-acetic acid-amido synthetase GH3.1; IAA-amino acid hydrolase ILR1), and catabolism (auxin peroxidases). Besides auxin-related genes, we also found genes responsive to ABA (e.g., protein EARLY-RESPONSIVE TO DEHYDRATION 7; E3 ubiquitin-protein ligase AIRP2) and ethylene (e.g., ethylene-overproduction protein 1; senescence-associated protein DIN1), and genes involved in detoxification (e.g., protein DETOXIFICATION 40) and response to ROS (e.g., protein ACTIVITY OF BC1 COMPLEX KINASE 7; aconitate hydratase 1) (Fig. 5). The detailed annotation of the SNPs and candidate genes can be found in the Supplementary Table S1.

**Table 2:** List of 50 significant SNPs detected by both XtX and association analyses and annotation of candidate genes flanking significant SNPs.

SNP	XtX	dB	REF	ALT	Location	Effect	Transcript	Description
chr1_14537036	24.47	39.57	G	T	intron	—	mRNA5872	protein SDA1 homolog
chr1_14537054	22.54	36.40	C	A	intron	—	mRNA5872	protein SDA1 homolog
chr1_14537074	21.97	38.67	A	C	intron	—	mRNA5872	protein SDA1 homolog
chr2_2760189	24.71	27.40	C	T	exon	Syn	mRNA24012	NEDD8-conjugating enzyme Ubc12
chr2_6395858	20.96	27.37	A	G	3′ UTR	—	mRNA27827	cytochrome P450 81E8-like
chr2_6417601	23.63	33.31	A	G	exon	Syn	mRNA27786	–NA–
chr2_6465577	20.81	28.79	T	G	3′ UTR	—	mRNA27788	probable beta-1,4-xylosyltransferase IRX14
chr2_6598346	20.77	30.39	T	A	intron	—	mRNA27796	DNA polymerase alpha catalytic subunit
chr2_6932804	22.25	26.66	T	C	exon	Syn	mRNA21680	BTB/POZ domain-containing protein NPY4-like
chr2_6933569	25.64	38.46	G	A	exon	Syn	mRNA21680	BTB/POZ domain-containing protein NPY4-like
chr2_10335789	20.75	24.83	A	G	exon	Syn	mRNA31749	probable serine/threonine-protein kinase At1g01540
chr2_10338020	21.75	30.09	T	C	3′ UTR	—	mRNA31749	probable serine/threonine-protein kinase At1g01540
chr2_10521233	24.67	44.53	G	A	intron	—	mRNA18714	nucleic acid binding protein
chr2_11103313	24.74	36.43	C	A	intron	—	mRNA22063	methionine gamma-lyase-like
chr2_11256523	33.19	51.56	A	C	intron	—	mRNA23765	ABC transporter F family member 3
chr2_11258472	27.42	40.30	G	A	intron	—	mRNA23765	ABC transporter F family member 3
chr2_11936871	36.79	56.94	T	C	3′ UTR	—	mRNA30083	homeobox-leucine zipper protein HAT4
chr2_12264640	24.82	34.56	G	C	exon	Missense	mRNA2446	lactoylglutathione lyase
chr2_12415913	22.91	31.12	A	G	intron	—	mRNA2457	CDK5RAP1-like protein
chr2_12471071	30.01	44.69	A	C	intron	—	mRNA2445	FG-GAP repeat-containing protein
chr2_12803074	24.98	31.02	T	C	5′ UTR	—	mRNA25976	NEP1-interacting protein 1
chr2_12842396	21.10	23.67	G	C	exon	Missense	mRNA25995	probable prolyl 4-hydroxylase 10
chr2_12842397	20.80	23.04	C	T	exon	Missense	mRNA25995	probable prolyl 4-hydroxylase 10
chr2_12912097	24.38	26.95	T	G	5′ UTR	—	mRNA25991	bifunctional nuclease 2 isoform X1
chr2_12944906	25.94	33.38	C	T	intron	—	mRNA25978	thiol-disulfide oxidoreductase LTO1
chr2_13304802	20.95	25.49	C	T	3′ UTR	—	mRNA36050	BTB/POZ and TAZ domain-containing protein 4-like
chr2_15907684	24.48	26.08	C	T	upstream	—	mRNA35013	proline-rich cell wall-like protein
chr2_15907767	27.04	33.23	C	T	upstream	—	mRNA35013	proline-rich cell wall-like protein
chr2_15907772	24.56	31.18	T	C	upstream	—	mRNA35013	proline-rich cell wall-like protein
chr2_16876599	35.05	53.82	A	G	exon	Syn	mRNA18763	E3 ubiquitin-protein ligase HOS1
chr2_16876687	35.09	50.60	C	T	exon	Missense	mRNA18763	E3 ubiquitin-protein ligase HOS1
chr2_16877118	34.64	48.77	C	T	exon	Syn	mRNA18763	E3 ubiquitin-protein ligase HOS1
chr2_16877152	35.93	50.17	G	C	exon	Missense	mRNA18763	E3 ubiquitin-protein ligase HOS1
chr2_16877205	33.18	56.73	A	G	exon	Syn	mRNA18763	E3 ubiquitin-protein ligase HOS1
chr2_16940118	37.15	62.17	T	C	exon	Syn	mRNA18740	phosphatidylserine decarboxylase proenzyme 1, mitochondrial
chr2_16940542	37.58	58.49	C	G	intron	—	mRNA18740	phosphatidylserine decarboxylase proenzyme 1, mitochondrial
chr2_17226080	21.00	29.68	C	T	intron	—	mRNA17848	neutral alpha-glucosidase
chr2_17481636	22.55	22.73	T	C	intron	—	mRNA40986	neutral alpha-glucosidase
chr2_19036813	23.15	32.59	C	G	intron	—	mRNA16552	lanC-like protein GCL1
chr2_19037918	23.54	29.22	A	G	intron	—	mRNA16552	lanC-like protein GCL1
chr3_20204819	24.78	39.25	G	T	exon	Missense	mRNA34528	vinorine synthase-like
chr3_22656106	31.21	44.51	T	A	exon	Syn	mRNA10875	vacuolar protein sorting-associated protein 35A
chr3_22656461	35.56	57.40	C	T	intron	—	mRNA10875	vacuolar protein sorting-associated protein 35A
chr3_22656485	29.48	42.70	A	T	intron	—	mRNA10875	vacuolar protein sorting-associated protein 35A
chr3_22658968	24.11	35.90	A	T	intron	—	mRNA10875	vacuolar protein sorting-associated protein 35A
chr3_22658999	30.84	44.53	T	G	exon	Syn	mRNA10875	vacuolar protein sorting-associated protein 35A
chr4_26530649	23.15	34.68	T	C	exon	Syn	mRNA5425	jmjC domain-containing protein 7
chr4_26530658	26.63	45.17	G	A	exon	Syn	mRNA5425	jmjC domain-containing protein 7
chr6_1272279	23.22	25.67	G	T	intron	—	mRNA8656	phosphoinositide phosphatase SAC7-like
chr7_6066346	25.80	45.31	C	T	exon	Syn	mRNA10279	glycosyl hydrolase family 43 protein

SNP names are represented by the chromosome and position.

## Discussion

In this study, we investigated genome-wide signatures of selection for 2,4-D herbicide resistance in red clover by contrasting Pool-Seq data from resistant and susceptible populations. Genomic studies to find regions associated with naturally-evolved resistance to 2,4-D have been largely unexplored. Elucidating the genetic and molecular basis of natural herbicide resistance is a central challenge for either developing resistant crops, improving herbicide targets, or predicting the potential of weeds to overcome herbicide mechanisms. Furthermore, we have also shown the feasibility of utilizing Capture-Seq technique, which in conjunction with the Pool-Seq approach, allowed the cost-effective identification of genetic variants. Therefore, this approach is also promising for similar genomics studies in non-model species with less resources.

The selective pressure imposed by 2,4-D treatment over multiple breeding cycles to obtain resistant cultivars has left genomic footprints of selection. In a preliminary screening, we detected allele frequency differences among pools with contrasting phenotypes. As part of breeding programs, the red clover synthetic cultivars used herein are connected by the pedigree and shared genetic relationship, as demonstrated by the covariance matrix and the PCA based on the allele frequencies. Therefore, to detect significant signatures of herbicide adaptation, we considered two other analytic methods that accounted for relatedness and also for sampling noise of Pool-Seq data. Taking these confounding factors into account, we detected 107 overly differentiated variants using the XtX statistics. Nonetheless, XtX is a covariable-free statistic that is powerful to identify SNPs subjected to a broader kind of adaptive constraint^36^. Therefore, XtX outlier loci can also be responding to a distinct selection pressure other than the herbicide. To refine the list of outlier loci, we considered a third strategy based on genome-wide association analysis using the resistant/susceptible phenotype as a population-specific covariable. From these combined approaches, we detected 50 SNPs exhibiting both strong genetic differentiation and significant association with the phenotype.

The 50 significant SNPs were located at six chromosomes, indicating that several genomic regions are putatively involved in the herbicide resistance adaptation. Resistance to herbicides with complex modes of action, such as 2,4-D, is indeed likely to be affected by many genes with minor-effects, arising gradually in the population via recombination of standing genetic variants into the same genetic background over generations^1,23,37,38^. The quantitative genetic architecture of 2,4-D resistance in red clover is also in agreement with the breeding strategy employed to obtain resistant plants, where recurrent cycles of mass selection under 2,4-D application were carried out to increase the number of favorable alleles in the population^12,30,39^. However, monogenic and dominant patterns of inheritance were inferred through segregation studies of the 2,4-D resistance phenotype in some weed species, such as wild mustard (*Brassica kaber* L.), prickly lettuce (*Lactuca serriola* L.), oriental mustard (*Sisymbrium orientale* L.), and wild radish (*Raphanus raphanistrum* L.)^40–44^. A single dominant resistance allele was also shown to be the causal basis for dicamba/2,4-D resistance in kochia^22^. In this sense, the consistent and higher number of significant variants detected through all approaches at chromosome 2 in red clover led us to speculate that a quantitative trait locus with major effect might exist in this region. Further studies are needed to draw this conclusion, but this result is already promising for marker-assisted selection in the red clover breeding program.

Most of the significant SNPs were located nearby or within protein-coding genes. However, the majority did not have a clear functional effect and their detection as outliers probably resulted from hitchhiking rather than a causative variation. Therefore, at this point, we cannot identify the specific loci and molecular mechanisms that directly contribute to the 2,4-D resistance adaptation. However, many interesting candidate genes are present at the vicinity of significant SNPs, providing some insights into potential mechanisms for 2,4-D resistance in red clover.

Out of the 50 significant SNPs, six were predicted to cause non-synonymous amino acid changes. Among those, two variants affected a gene likely encoding an E3 ubiquitin-protein ligase HOS1. In *Arabidopsis*, HOS1 mediates the proteasomal degradation of ICE1, which is a transcription factor involved in chilling and freezing tolerance^45^. Interestingly, an ICE1-homolog was upregulated by 2,4-D in resistant but not in susceptible populations of wild radish^29^, suggesting that the regulation of ICE1 may also influence 2,4-D stress tolerance. Moreover, HOS1 is also required for photoperiodic control of flowering in *Arabidopsis*, with distinct *hos1* loss-of-function mutants displaying an early flowering phenotype^46^. Although there was no selection for early flowering in the development of the first 2,4-D resistant cultivar, ‘FL24D’ grew earlier in spring than any other red clover cultivar at the University of Florida^12^. It seems plausible that the early flowering time in red clover resulted from a pleiotropic effect or genetic hitchhiking of *hos1* or other regulators of flowering time along with 2,4-D resistance locus.

To explore the possibility that selection targeted untyped variants in the region flanking significant SNPs, we also annotated the genes within a ±100 kb window. Based on sequence homology, we detected several candidate genes with putative orthologs known to be directly involved in auxin homeostasis, such as regulators of auxin response, transport, conjugation, and catabolism. Besides auxin-related genes, we also found genes responsive to ABA and ethylene, and genes involved in detoxification and response to ROS that may constitute NTSR mechanisms. We also highlighted genes encoding cytochrome P450 family members as they have been identified as potential mediators of rapid detoxification mechanism for different classes of herbicides^47–50^, including auxinic herbicides, such as quinclorac^51^ and potentially 2,4-D^27,28^. Although the aforementioned genes seem to have a plausible role in the auxin related pathways and stress responses, more studies and functional validation experiments are needed.

In summary, by using a cost-effective approach, we were able to identify genomic regions, mainly at chromosome 2, that likely contain the gene(s) responsible for 2,4-D resistance adaptation in red clover. We believe that our findings provided a promising starting point for marker-assisted selection implementation in the red clover breeding program and for guiding the discovery of novel auxinic herbicide resistance mechanisms.

## Methods

### Plant material

Red clover cultivars are synthetic populations generated by open-pollination of selected parents and propagated for a limited number of generations. In this study, six resistant and four susceptible pools of individuals from synthetic red clover populations were used (Fig. 1). The red clover synthetic cultivar, ‘FL24D,’ was specifically bred for 2,4-D tolerance^30^. ‘FL24D’ was generated after six cycles (Gen6) of phenotypic recurrent selection using a source germplasm (Gen0) of three different commercial synthetic cultivars (‘Kenstar’, ‘Nolins Red’, and ‘Cherokee’) with 2,4-D treatment as selection factor at the University of Florida. A detailed description of how the ‘FL24D’ cultivar was generated can be found in Quesenberry *et al*.^30^. Briefly, seedlings derived from the intercross of the Gen0 population were sprayed with 1.1 kg a.i. ha-1 of 2,4-D dimethylamine salt formulation, and resprayed using similar rates three weeks later. Plants with superior survival and regrowth were intercrossed. This process was repeated throughout six cycles and the resistant synthetic cultivar ‘FL24D’ was obtained (Fig. 1). The resistance level of ‘FL24D’ was compared against a susceptible cultivar ‘Southern Belle’ in greenhouse and field experiments under three rates of 2,4-D (1/2x = 0.53 kg ha^−1^, 1x = 1.06 kg ha^−1^, and 2x = 2.12 kg ha^−1^). A damage rating scale of 1-to-9 was used, where 9 meant no visible symptoms and 1 meant severe leaf and stem curling and/or plant death. For example, ‘FL24D’ rated 7.0 whereas ‘Southern Belle’ rated 1.2 at the 1x concentration in greenhouse experiment^30^.

Individuals from the ‘FL24D’ cultivar were used as one of the resistant pools (Gen6). Individuals from the three cultivars (‘Kenstar,’ ‘Nolins Red,’ and ‘Cherokee’) that make up the foundational germplasm were used as the initially herbicide‐susceptible population in our experiment, in a pool sample named Gen0. Additionally, the cultivar ‘Cherokee,’ which is one of the parents from the initial germplasm with earlier spring growth, and ‘Southern Belle,’ a cultivar developed from ‘Cherokee’ for root-knot nematode resistance^52^, were also included as 2,4-D susceptible populations.

As ‘FL24D’ is a synthetic cultivar, genetic and phenotypic variability exist among the individuals from that cultivar. To increase the chances of including only highly resistant individuals, another 2,4-D application was performed on the ‘FL24D’ population, and individuals with minor damage were selected to compose the ‘FL24DElite’ pool (Fig. 1). ‘FL24D’ individuals were also split into early flowering ‘Early24D’ and late flowering ‘Late24D’ pools. Furthermore, ‘FL24D’ was also introduced into the breeding program of northern adapted red clover at the University of Kentucky^39^. The 2,4-D resistant line, ‘UK,’ was developed after eight recurrent selection cycles, using ‘FL24D’ and the susceptible cultivar ‘Kenland’ as parents. Similar to the way that ‘FL24DElite’ was generated, the pool ‘UKElite’ was created from ‘UK.’ The susceptible parental cultivar, ‘Kenland’, was also included in the analyses. More details on how cultivars were developed can be obtained at^30,39,52^.

### Total genomic DNA extraction

One hundred seeds from each population were germinated in petri-dishes. Out of those, 72 germinated seeds from each population were transplanted individually into 5-cm-square trays containing an equal mixture of local fine sand and potting mix. Seedlings were grown in a greenhouse. Young trifoliate leaves were collected 28 days after seed germination. Total genomic DNA was extracted from leaves of each individual plant using the DNeasy Plant Mini Kits (Qiagen, Valencia, CA, USA). DNA quality and purity were assessed on 1% agarose gel electrophoresis and by the A260/280 ratio, using a Nanodrop 1000 spectrophotometer (Thermo Scientific, Wilmington, DE, USA). DNA was accurately quantified using a Qubit Fluorometer (Invitrogen, Carlsbad, CA, USA) with the PicoGreen dsDNA Assay Kit (Molecular Probes, Eugene, OR, USA). DNA samples from the same population, with satisfactory quality and quantity, were mixed in an equimolar concentration to generate a DNA pool for sequencing (Table 1).

### Probe design and genotyping of pooled samples

Genotyping of DNA pools using next generation sequencing (Pool-Seq) was carried out by RAPiD Genomics (Gainesville, Florida, USA) using a sequence capture approach (Capture-Seq). Briefly, 120-mers probes were designed based on publicly available expressed sequence tags (ESTs) and assembled transcripts from the closely related species white clover (*Trifolium repens* L.)^53^. EST sequences (15,260) and transcript sequences (71,545) were filtered to remove identical and low-quality sequences using SeqClean^54^. Filtered sequences were aligned to the *Medicago truncatula* L. genome^55^ and to the ‘Milvus B’ red clover genome^56^, resulting in an average of 87.65% and 92.83% similarity, respectively. To synthesize biotinylated oligonucleotide probes for Capture-Seq genotyping, 15,885 sequences that aligned to the genome of both species were selected, avoiding mitochondrial and chloroplast DNA, enriching for exonic sequences, with GC content between 20–60%, and lacking homopolymers (less than eight nucleotides).

Ten sequencing libraries from pooled DNA samples were prepared according to Neiman *et al*.^57^. Sequencing was carried out in two batches. The first samples were sequenced using the Illumina NextSeq. 500 platform with 75 bp paired-end cycles. The second sequencing batch was performed using the Illumina HiSeq. 3000 with 100 bp paired-end cycles (Table 1).

### SNP calling and filtering

Raw reads were trimmed by quality using Trimmomatic v.0.36^58^ with the parameters “TRAILING:3 SLIDINGWINDOW:4:15 MINLEN:50.” The software BWA v.0.7.17^59^ was used to align the trimmed paired reads to the seven linkage groups (“chr”) that compose the red clover reference genome ‘Milvus B’ (2n = 2x = 14) (Accession Number: GCA_900079335.1)^56^. Alignment files were converted into bam files and sorted using SAMtools v. 1.3.1^60^. Picard Tools v. 2.18.3 was used to remove PCR duplicates with the “MarkDuplicates” function (http://broadinstitute.github.io/picard/). SNP calling was performed with the software FreeBayes v1.0.2 software^61^. SNPs were further filtered by selection for those with: (i) minimum mapping quality of 20; (ii) only biallelic locus; (iii) no missing data; (iv) minimum depth of coverage of 40; and (v) maximum depth of coverage of 400 (corresponding to the lowest 95th percentile value of the empirical coverage distribution across the pooled samples).

### Genetic relationship among the pooled samples

To assess the genetic relationship among each pair of pooled samples, we estimated the scaled covariance matrix of allele frequencies (Ω) using the software Baypass v.2.1 under the core model^36^. The Ω matrix was transformed into a correlation matrix, using the cov2cor() R function and a heatmap was generated using the corrplot() function from the R package corrplot. A principal component analysis (PCA) was carried out based on the Ω matrix with the dudi.pca() function of the R package ade4^62^.

### Pairwise differences in allele frequency

As a preliminary screening for changes in allele frequency potentially related to the selection pressure toward herbicide resistant in red clover, we contrasted the differences in raw allele frequencies for each SNP among all ten pooled samples. Alternative and reference read counts were extracted from the variant calling file using VCFtools v.0.1.15^63^. Alternative allele frequency was estimated for each locus within the pool by dividing the alternative read count by the total read count (i.e., alternative plus reference read counts). The absolute pairwise differences in allele frequency among pools were plotted with a threshold value of 99.9^th^ percentile of the allele frequency difference distribution across all values.

### Genome scan for adaptive differentiation

SNPs subjected to adaptive differentiation were formally inferred through the XtX differentiation measure^64^. The XtX statistic is analogous to F_*ST*_, but explicitly accounts for the relationship among populations and sampling noise in pooled samples (variation in sequencing depth across populations and SNPs)^64^. The XtX genetic differentiation value for each SNP was estimated under the core model implemented in Baypass v.2.1^36^. To run the Baypass core model, we used the alternative and reference read count data for the ten pools and the haploid pool sizes as inputs, with the –d0yij option set at 8 for Pool-Seq mode and MCMC options as 25 short pilot runs (1,000 iterations each) to adjust the proposal distributions for each model parameter. Subsequently, an 100,000 burn‐in period and an 100,000 updating steps were performed with a thinning interval of 40 steps. A pseudo‐observed data set (POD) was simulated considering the same parameters as those estimated in the original data using the R function simulate.baypass() available in the BayPass software. The POD was further analyzed under the core model with the same parameters to estimate the posterior predictive distribution of the XtX statistics under neutrality. We compared the posterior estimates of Ω in the simulated data against the original data using FMD distance^65^ to assess the precision and robustness of the simulated data. The 99.9^th^ percentile of this empirical distribution was used to calibrate the original XtX values, i.e., the POD analysis provided the 0.1% threshold XtX value as a decision criterion for discriminating between selection and neutrality, and detect overly differentiated SNPs^36^.

### Association analysis with the 2,4-D resistance/susceptibility phenotype

In order to refine the list of outlier loci, we also performed a genome‐wide association analysis using the herbicide susceptibility or resistance as a population‐specific covariable (coded as a binary variable with values of −1 and 1, respectively). The read count data was analyzed under the auxiliary variable covariate (AUX) model, also implemented in Baypass v.2.1^36^. In the AUX model, for each SNP *i* and a given covariable *k*, a Bayesian (binary) auxiliary variable *δ*_*ik*_ is attached to the regression coefficient *β*_*ik*_ in a model that also accounts for relatedness using the Ω matrix and sampling noise. The binary auxiliary variable indicates whether a specific SNP can be regarded as associated with the covariable *k* (*δ*_*ik*_ = 1) or not (*δ*_*ik*_ = 0). Therefore, the posterior mean of *δ*_*ik*_ can be interpreted as a posterior probability of association of the SNP *i* with the covariable *k*, from which a Bayes factor (BF) is derived, also taking multiple-testing issues into account^36^. The same MCMC parameters specified in the core model were also used for running the AUX model. The BF was further converted in deciban (dB) units using the transformation 10 log_10_(BF). Considering the Jeffreys’ rule to quantify the strength of evidence^66^, we set dB = 20 as a stringent threshold for “decisive evidence.”

### Candidate gene mining

SNPs consistently detected across the latter two approaches, i.e., SNPs overly differentiated at XtX >1% POD significance threshold and association at the 20-dB threshold, were further considered as candidates for evaluation. The genomic position and functional effect of significant SNPs were annotated using snpEff v.4.3^67^, using the ‘Milvus B’ genome and gene predictions^56^. Predicted gene models were retrieved from the Ensembl Plants database^68^. To explore the possibility that selection targeted untyped variants in physical linkage with significant SNPs, we defined an *ad hoc* window of ±100 kb surrounding significant SNPs and annotated all genes within this interval. Gene annotations were performed using the Blast2GO tool with BLASTp searching against the non-redundant protein database^69^. Additional information about the potential role of candidate genes was recovered from SWISS-PROT curated annotations^70^.

### SNP validation

The most significant variant at each chromosome was selected for SNP validation in an independent set of individuals from the resistant cultivar ‘FL24D’ and the susceptible cultivar ‘Southern Belle’. The two SNPs causing non-synonymous mutations at the putative *hos1* gene at chromosome 2 were selected, plus one SNP at each chromosome 1, 3, 4, 6, and 7. Primers were designed in the region surrounding the SNPs (Supplementary Table S2). Genomic DNA from ten individuals of each cultivar were extracted and used as template in PCR amplifications. Amplifications were carried out using Kapa Hifi Hotstart DNA polymerase (Kapa Biosystems, Boston, MA, USA), with the following thermal cycling conditions: 95 °C for 3 min, 30 cycles of 98 °C (20 s), 58 °C (15 s), 72 °C (20 s), and a final extension of 72 °C for 1 min. Amplicons were visualized on 1% agarose gel prior to PCR clean-up and Sanger sequencing at Genewiz Corporation (South Plainfield, NJ, USA). Sequences were processed and aligned using CLC genomics workbench v12. Fisher’s exact test was performed using the R software (http://www.r-project.org).

## Supplementary information

Supplementary Information

Supplementary Information 2

## Data Availability

Raw sequence data for each pooled sample were deposited in NCBI’s sequence read archive (SRA) under accession numbers from SRR8157534 to SRR8157543. The corresponding Pool-Seq libraries are provided in Table 1.
