# Supplementary material for: Cost-effective detection of genome-wide signatures for 2,4-D herbicide resistance adaptation in red clover
Source: Sci Rep. 2019 Dec 27;9:20037. doi: 10.1038/s41598-019-55676-9 (PMC6934753; doi:10.1038/s41598-019-55676-9)
Supplement: Supplementary file 2 — Supplementary Information 2 [file 41598_2019_55676_MOESM2_ESM.pdf]

Supplementary Figure 1

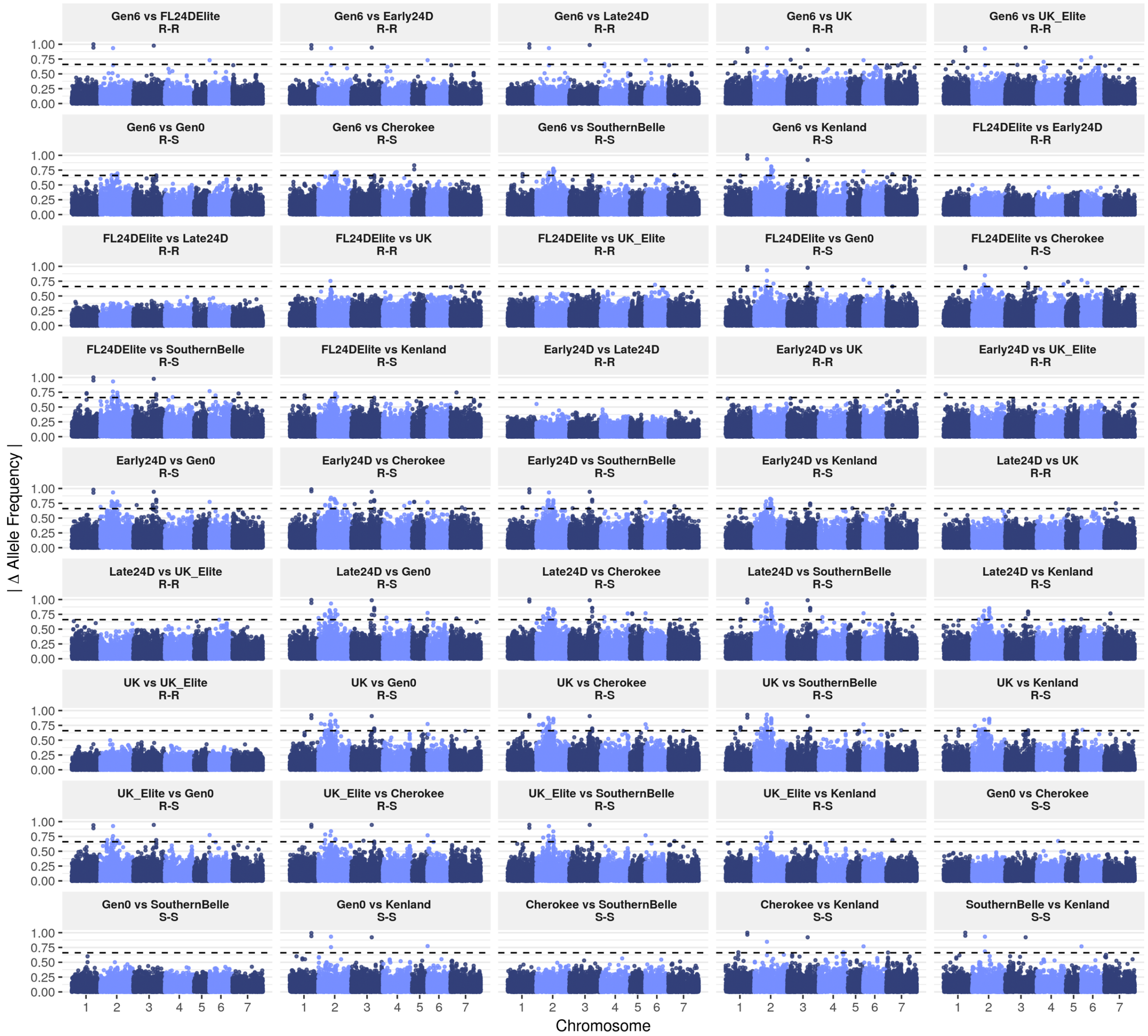

Supplementary Figure 1. Pairwise allele frequency differences of 11,768 SNPs among the ten pools of synthetic cultivars. Absolute values were plotted. Resistant-Susceptible, Resistant-Resistant, and Susceptible-Susceptible comparisons are indicated by R-S, R-R, and S-S, respectively. The threshold was determined as the 99.9th percentile of the allele frequency difference distribution across all values.

Supplementary Table 2

Supplementary Table 2. Primers designed for amplification and sequencing of regions harboring significant SNPs.

SNP_ID	CHR_POSITION	PrimerF	PrimerR	Fragment size	REF	ALT
SNP1	chr1_14537036	5'-TTCAGTCGTGTCTTCGTC-3'	5'-CTTGCTTTCATCCATCGT-3'	210	G	T
SNP2	chr2_16876687	5'-GGAACAACTAAAAAGCGGAA-3'	5'-CAGTCTACACTCTACCCAA-3'	781	C	T
SNP3	chr2_16877152	5' GGAACAACTAAAAAGCGGAA-3'	5'-CAGTCTACACTCTACCCAA-3'	781	G	C
SNP4	chr3_22656461	5'-CTTCGTTGCCTTCTTGTC-3'	5'-CTCTTCTTACCTTCACTCTT-3'	436	C	T
SNP5	chr4_26530658	5'-TCACCACAAACAACAGCA-3'	5'-CATCACCCCAAACAAACC-3'	462	G	A
SNP6	chr6_1272279	5'-TCTGGTTCTTCTTTGGCT-3'	5'-CCTCAATATCCCAACAACAC-3'	277	G	T
SNP7	chr7_6066346	5'-GTGTTCTTTCCTGGTCGT-3'	5'-TGCCATGTTTCCAATCGT-3'	375	C	T

Supplementary Figure 2

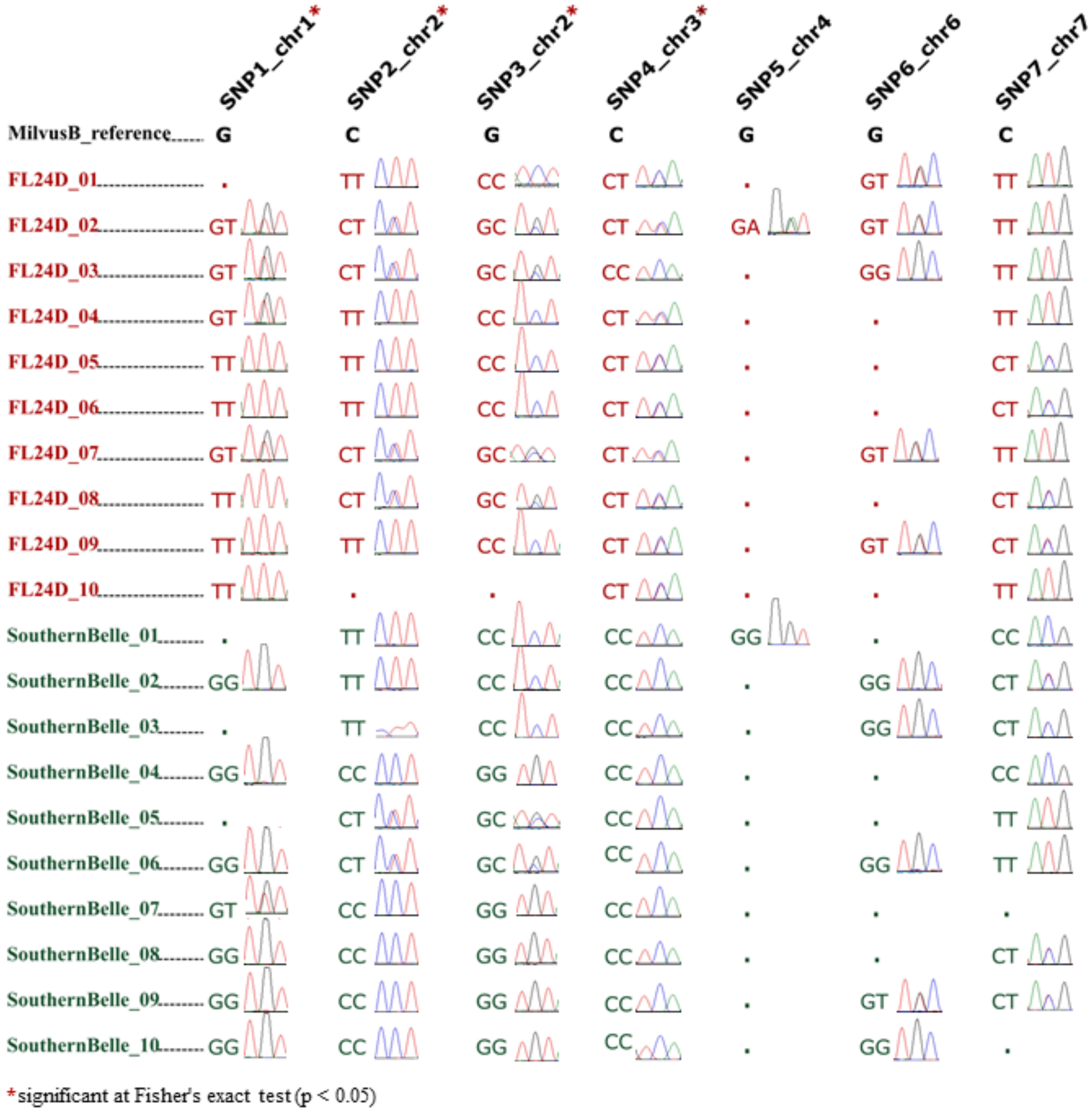

Supplementary Figure 2. Genotypes of an independent set of individuals for seven significant SNPs for 2,4-D resistance located at distinct chromosomes. Susceptible individuals from 'Southern Belle' cultivar are indicated in green, while resistant individuals from 'FL24D' cultivar are indicated in red. Sanger chromatograms are shown for each genotype, where the middle peak harbors the SNP. Dots indicate failed sequencing.
